# The prognosis was poorer in colorectal cancers that expressed both VEGF and PROK1(No correlation coefficient between VEGF and PROK1)

**DOI:** 10.18632/oncotarget.4744

**Published:** 2015-07-30

**Authors:** Takanori Goi, Toshiyuki Nakazawa, Yasuo Hirono, Akio Yamaguchi

**Affiliations:** ^1^ First Department of Surgery, University of Fukui, 9101193, Japan

**Keywords:** colorectal cancer, prokineticin1(PROK1), vascular endothelial growth factor (VEGF)

## Abstract

The angiogenic proteins vascular endothelial growth factor (VEGF) and prokineticin1 (PROK1) proteins are considered important in colorectal cancer, the relationship between their simultaneous expression and prognosis was investigated in the present study.

VEGF and PROK1 expression in 620 primary human colorectal cancer lesions was confirmed via immunohistochemical staining with anti-VEGF and anti-PROK1 antibodies, and the correlation between the expression of these 2 proteins and recurrence/prognosis were investigated.

VEGF protein was expressed in 329 (53.1%) and PROK1 protein was expressed in 223 (36.0%). PROK1 and VEGF were simultaneously expressed in 116 (18.7%) of the 620 cases. The correlation coefficient between VEGF expression and PROK1 expression was *r* = 0.11, and therefore correlation was not observed. Clinical pathology revealed that substantially lymphnode matastasis, hematogenous metastasis, or TMN advanced-stageIV was significantly more prevalent in cases that expressed both VEGF and PROK1 than in the cases negative for both proteins or those positive for only 1 of the proteins.

Also the cases positive for both proteins exhibited the worst recurrence and prognosis. In the Cox proportional hazards model, VEGF and PROK1 expression was an independent prognostic factor.

The prognosis was poorer in colorectal cancers that expressed both PROK1 and VEGF relative to the cases that expressed only 1 protein, and the expression of both proteins was found to be an independent prognostic factor.

## INTRODUCTION

The therapeutic outcomes are relatively favorable for early-stage colorectal cancer but unsatisfactory for advanced (stage III/IV) cases, a phenomenon that requires further investigation [1]. Generally, the determining prognostic factor in colorectal cancer is hematogenous metastasis, including liver metastasis [1]. Elucidation of the metastatic mechanism is considered important and will provide the initial step toward the development of novel therapies.

Various molecular biological investigations of hematogenous mechanisms have revealed the following process in which angiogenic factors are closely involved during multiple steps [2–5]: cancer cells dissociate from the primary lesion, followed by basement membrane disintegration, movement within the interstitium, vascular invasion, and cancer cell adhesion, invasion, and proliferation in the target organ [6]. Oxygen and nutrition are supplied to tumor cells from the blood vessels and once the tumor has exceeded 1 mm in size, the blood vessels become hypoxic and angiogenesis becomes an absolute necessity [7, 8]. Cancer cells can directly produce angiogenic growth factors or proteases and release extracellular matrix (ECM)-bound vascular endothelial growth factor (VEGF) to stimulate the vessels and induce angiogenesis [9–10]. VEGF-A was identified as an angiogenic factor in 1989, and the family members VEGF-B, C, and D were subsequently isolated [11–13]. VEGF-A is a 45-kD dimeric protein; in humans, VEGF121, VEGF165, VEGF189, and VEGF206 are produced via alternative splicing. In particular, VEGF165 is thought to be expressed in both healthy and tumor tissues [14–15]. In cancer tissues, VEGF165 correlates strongly with hematogenous metastasis in gastric cancer, colorectal cancer, lung cancer, and other malignant tumors [16–19]. The current National Comprehensive Cancer Network (NCCN) guidelines include anti-VEGF antibody therapy in addition to systemic FOLFOX (folinic acid/5-fluorouracil/oxaliplatin) and FOLFIRI (folinic acid/5-fluorouracil/irinotecan) chemotherapy for the treatment of unresectable/recurrent colorectal cancer [20].

Ferrara reported that Prokineticin 1 (PROK1) acts as a vascular endothelial growth factor in the adrenal gland, ovary, testis, and other endocrine tissues [21]. According to subsequent reports, the post-resection prognosis was significantly poorer for colorectal cancer patients with positive PROK1 mRNA expression than for PROK1-negative patients, and PROK1 protein was found to be involved in angiogenesis and lung metastasis from colorectal cancers [22, 23]. According to recent findings, an anti-PROK1 antibody suppressed angiogenesis in a PROK1 receptor-expressing colorectal cancer subset, and the infiltrative ability of these cells was promoted by an autocrine PROK1 mechanism [24]. PROK1 expression also reportedly correlated with cancer progression and metastasis in pancreatic duct cancer, prostate cancer, neuroblastoma, and several other malignant tumors [25–28]. Therefore, PROK1 is a significant factor associated with tumor malignancy.

To date, individual angiogenic proteins have been investigated as important factors related to hematogenous metastasis in colorectal cancer; however, no study has evaluated 2 angiogenic proteins simultaneously. Accordingly, we examined both VEGF and PROK1, which have been independently correlated with the hematogenous metastasis of colorectal cancer, and obtained the interesting results reported below.

## RESULTS

### VEGF and PROK1 protein expression in human colorectal cancer tissues

**Figure 1:**
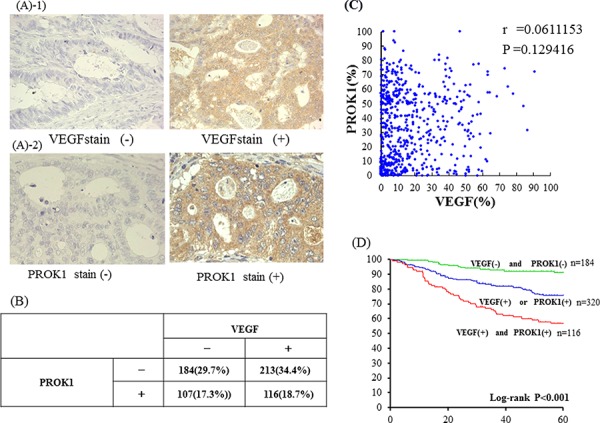
A-1. Immunohistochemical staining with anti-VEGF mAb Left: VEGF expression was not detected inprimary colorectal cancer lesion Right: VEGF expression was detected in primary colorectal cancer lesions. **A-2.** Immunohistochemical staining with anti-PROK1 mAb Left: PROK1 expression was not detected inprimary colorectal cancer lesion. Right: PROK1 expression was detected in primary colorectal cancer lesions. **B.** The relationship between VEGF expression and PROK1 expression in sporadic 620 patients with primary colorectal cancer VEGF expression was observed in 325 (52.5%) of the 620 cases, whereas PROK1 was expressed in 223 (36.0%) of the 620 cases. Neither VEGF nor PROK1 was expressed in 184 (30.1%) of the 620 cases. Both proteins were expressed in 115 (18.6%) of the 620 cases VEGF alone was expressed in 210 (33.9%) of the 620 cases, and PROK1 alone was expressed in 108 (17.4%) of the cases. **C.** The correlation between VEGF expression and PROK1 expression The correlation coefficient between VEGF expression and PROK1 expression was *r* = 0.11. Correlation was not observed. **D.** Relationship between PROK1/VEGF expression and survival rate in colorectal cancer patients. The 5-year survival rate was 91.3% in the colorectal cancer patients with expression of neither VEGF nor PROK1, 76.4% with expression of either VEGF or PROK1; and 57.3% with expression of both VEGF and PROK1.

The Fig. 1A shows representative cases of positive expression in primary human colorectal cancer lesions. VEGF expression was observed in 329 (53.1%) of the 620 cases, whereas PROK1 was expressed in 223 (36.0%) of the 620 cases.

### The relationship between VEGF expression and PROK1 expression in human colorectal cancer tissues

Neither VEGF nor PROK1 was expressed in 184 (29.7%) of the 620 cases. VEGF alone was expressed in 213 (34.4%) of the 620 cases, and PROK1 alone was expressed in 107 (17.3%) of the cases. Both proteins were expressed in 116 (18.7%) of the 620 cases (Fig. 1B). The correlation coefficient between VEGF expression and PROK1 expression was *r* = 0.11, and therefore correlation was not observed (Fig. 1C).

### The relationship between PROK1/VEGF expression in human colorectal cancer tissues and the survival rate

The 5-year survival rates for all colorectal cancer patients were 91.3% among cases with no VEGF/PROK1 expression, 76.4% among cases that expressed either protein, and 57.3% among cases that expressed both proteins; the latter rate indicated a significantly poorer prognosis (Fig. 1D).

### The relationship between PROK1 and VEGF protein expression and clinicopathologic factors

An investigation of the relationship between VEGF/PROK1 protein expression and clinicopathological factors revealed that the group that expressed either of the angiogenic proteins included a significantly greater number of cases with a substantially lymphnode metastasis and hematogenous metastasis(TMN advanced-stage III, IV) relative to the group lacking the expression of both proteins. Furthermore, the group that expressed both proteins had a significantly greater number of cases with lymphnode metastasis, hematogenous metastasis, and TMN advanced-stageIV tumors relative to the other 2 groups. No relationships were found with respect to gender, age, histological type, histological type, lymphatic invasion, venous invasion, and peritoneal metastasis (Table 1).

**Table 1:** Correlation between clinicopathologic features and VEGF and PROK1 expression

	Total	VEGF and PROK1	VEGF or PROK1	VEGF and PROK 1	
		No. of negative cases	No. of positive cases	No. of positive cases	*P*-value
All cases	620	184	320	116	
Gender					0.740
Male	365	107	186	72	
Female	255	77	134	44	
Age (average 66.5)					0.962
<55	108	31	6	21	
≧55 to <65	143	39	74	30	
≧65 to <75	187	57	95	35	
≧75	182	57	95	30	
Histological type					0.313
Well+Mode	572	168(91.4%)	300(93.8%)	104(89.7%)	
Poor	28	8(4.3%)	11(3.4%)	9(7.8%)	
Mucinous	20	8(4.3%)	9(2.8%)	3(2.5%)	
Serosal invasion					0.067
Negative	148	55(29.9%)	70(21.9%)	23(19.8%)	
Positive	472	129(70.1%)	250(78.1%)	93(80.2%)	
Lymphatic invasion					0.007
Negative	99	42(22.8%)	39(12.2%)	18(15.5%)	
Positive	521	142(77.2%)	281(87.8%)	98(84.5%)	
Venous invasion					0.946
Negative	244	74(40.2%)	124(38.7%)	46(39.7%)	
Positive	376	110(59.8%)	196(61.3%)	70(60.3%)	
Peritoneal metastasis					0.330
Negative	600	181(98.4%)	308(96.2%)	111(95.7%)	
Positive	20	3(1.6%)	12(3.8%)	5(4.3%)	
Hematogenous Metastasis					<0.001
Negative	560	178(96.7%)	293(91.6%)	89(76.7%)	
Positive	60	6(3.3%)	27(8.4%)	27(23.3%)	
		*p* = 0.024 *p* < 0.001			
T(TNM 6th)					0.067
T1–2	148	55(29.9%)	70(21.9%)	23(19.8%)	
T3–4	472	129(70.1%)	250(78.1%)	93(80.2%)	
N(TNM 6th)					<0.001
N0	330	121(65.8%)	164(51.3%)	45(38.8%)	
N1–2	290	63(34.2%)	156(48.7%)	71(61.2%)	
		*p* = 0.021 *p* = 0.021			
Stage(TNM 6th)					<0.001
I	117	49(26.6%)	53(16.6%)	15(12.9%)	
II A	114	40(21.8%)	58(18.1%)	16(13.8%)	
II B	85	35(19.0%)	42(13.1%)	8(6.9%)	
III A	19	5(2.7%)	10(3.1%)	4(3.4%)	
III B	130	30(16.3%)	77(24.1%)	23(19.8%)	
III C	74	18(9.8%)	39(12.2%)	17 (14.7%)	
IV	81	7(3.8%)	41(12.8%)	33(28.5%)	
		*P* < 0.001 *p* < 0.001			

Well, well differentiated adenocarcinoma; Mode, moderately differentiated adenocarcinoma; Poor, poorly differentiated adenocarcinoma; Muc, mucinous adenocarcinoma.

### The relationship between both PROK1 and VEGF proteins expression and the hematogenous metastatic recurrence rate according to the colorectal cancer stage

Among stage III colorectal cancers, the hematogenous metastatic recurrence rates were 11.3% among cases with no expression of the 2 proteins, 24.6% among cases that expressed either protein, and 34.1%, a significant increase, among cases that expressed both proteins (Table 2). Among stage I and II cancers, no significant differences were observed in the hematogenous metastatic recurrence rates among the groups that did and did not express VEGF and/or PROK1.

**Table 2:** Recurrence rate of hematogenous metastasis according to VEGF and PROK 1 expression in each stage of colorectal cancers

	VEGF, PROK1		VEGF or PROK1		VEGF and PROK1			
Stage	negative		positive		positive			
Grouping	cases recurrence(%)		cases recurrence(%)		cases recurrence(%)			*P*
All cases	177	12 (6.8%)	279	44 (15.8%)	83	19	(22.9%)	0.001
I	49	2 (4.1%)	53	2 (3.8%)	15	1	(6.7%)	0.884
II	75	4 (5.3%)	100	11 (11%)	24	3	(12.5%)	0.369
III	53	6 (11.3%)	126	31 (24.6%)	44	15	(34.1%)	0.027

### The relationship between both PROK1 and VEGF expression in human colorectal cancer tissues and the survival rate

**Figure 2:**
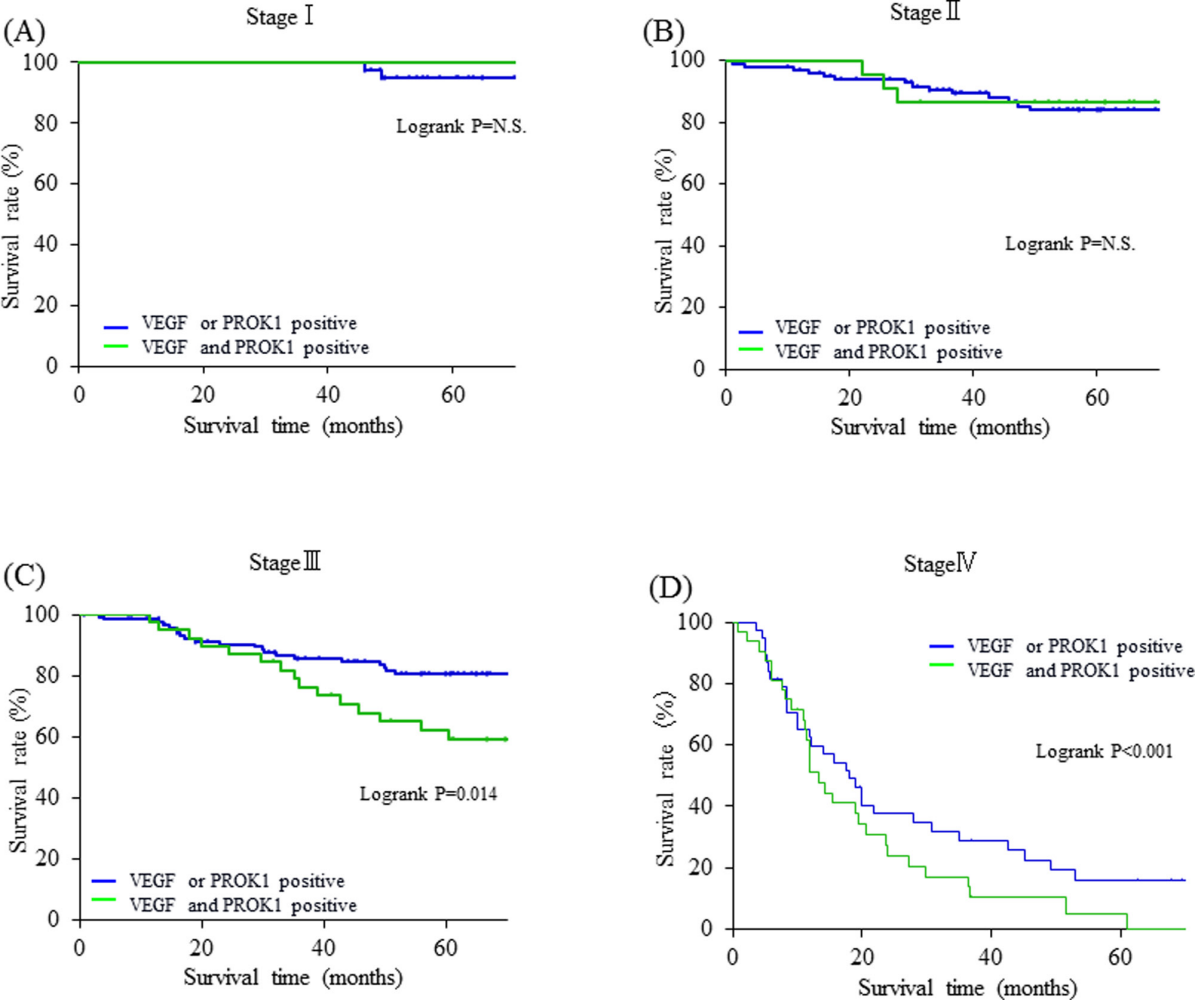
Relationship between the expression of VEGF/PROK1 protein and survival rates in colorectal cancer patients **A.** Stage I. **B.** Stage II. **C.** Stage III. **D.** Stage IV. For Stage III and Stage IV colorectal cancer patients, Patients with both VEGF and PROK1 expressions tumors had significantly poorer prognosis than those with either VEGF or PROK1 expression tumors.

The 5-year survival rates for Stage III colorectal cancer patients were 84.4% among cases that expressed either VEGF or PROK1 protein, and 59.1% among cases that expressed both proteins (Fig. 2C). For Stage IV colorectal cancer patients, the 5-year survival rate was 16.1% among cases that expressed either VEGF or PROK1 protein and 5.1% among cases that expressed both proteins (Fig. 2D). Therefore, for both stages, the survival rates were significantly lower in cases with both PROK1 and VEGF expression. No significant differences in survival were observed between patients with Stage I and Stage II colorectal cancer in terms of both VEGF and PROK1 expression in the primary lesions (Fig. 2A, 2B).

### Prognostic factors in colorectal cancer patients

The univariate analysis, conducted according to a Cox proportional hazards model, revealed that both PROK1 and VEGF protein expression, histological type, serosal invasion, lymphatic invasion, venous invasion, lymphnode metastasis, peritoneal metastasis, and hematogenous metastasis were factors that significantly correlated with prognosis. The multivariate analysis indicated that both VEGF and PROK1 protein expression, histological type, serosal invasion, lymphnode metastasis, and hematogenous metastasis were independent prognostic factors. The hazard ratio for VEGF/PROK1 protein expression was 2.317 (Table 3).

**Table 3:** Pathological findings and PROK1/VEGF as prognostic factor for colorectal cancer patients

	Univariate analysis			Multivariate analysis		
	Hazard Ratio	95% CI	*P*-value	Hazard Ratio	95% CI	*P*-value
Gender	0.735	0.520–1.041	0.083			
Age	0.999	0.985–1.013	0.589			
PROK1+VEGF	3.316	2.364–4.652	0.000	2.317	1.618–3.317	0.000
Histological type	2.168	1.669–2.816	0.000	2.091	1.583–2.761	0.000
(Well^a^+Mode^b^ /Poor^c^/Muc^d^)						
Serosal invasion	5.931	2.905–12.107	0.000	3.758	1.666–8.479	0.001
Lymphatic invasion	3.057	1.556–6.008	0.001	1.181	0.570–2.448	0.655
Venous invasion	1.736	1.209–2.493	0.003	0.973	0.657–1.440	0.891
Lymphnode metastasis	3.193	2.221–4.592	0.000	1.686	1.141–2.490	0.009
Peritoneal metastasis	5.001	2.754–9.080	0.000	1.097	0.579–2.078	0.776
Hematogenous	13.544	9.439–19.434	0.000	8.808	5.883–13.189	0.000
Metastasis						

a Well, well differentiated adenocarcinoma

b Mode, moderately differentiated adenocarcinoma

c Poor, poorly differentiated adenocarcinoma

d Muc, mucinous adenocarcinoma.

## DISCUSSION

In recent years, gastrointestinal cancer and other various malignant tumors have been investigated via molecular biological techniques, and a considerable number of genes are thought to be involved in the metastatic and growth mechanisms [29, 30]. In particular, a number of reports have implicated angiogenic growth factor involvement in hematogenous metastasis, the significance of which is apparently considerable [31, 32]. Among various gastrointestinal tumors, the most well known VEGF was found to correlate with the hematogenous metastasis of gastric and colorectal cancers [16–18], and elevated VEGF expression induced angiogenesis near the tumor. Furthermore, increased VEGF expression was reported to be a poor prognostic factor for colorectal cancer, among other cancers.

Although the PROK1 protein investigated in the present study was identified as an angiogenic factor in endocrine cells by Ferrara [21], the researchers observed its involvement in the hematogenous metastasis and autocrine mechanism-based invasive ability of human colorectal cancer cells, and its significance in various malignant tumors was demonstrated via its relationship with malignancy in prostate cancer, neuroblastoma, and pancreatic duct cancer [25–28]. Cancer cell growth or progression is considered consequent to the overexpression or deficiency of various factors and, as mentioned earlier, no reports have been published on the simultaneous investigation of VEGF and PROK1, both of which are substantially involved in the hematogenous metastasis of colorectal cancer. Therefore, the simultaneous investigation of these 2 factors is significant.

According to the study results, both PROK1 and VEGF were expressed in approximately 20% of the tumors, either PROK1 or VEGF in approximately 50%, and neither factor in approximately 30%. Clinicopathologically, both PROK1 and VEGF expression relative to the groups lacking the expression of both proteins or either VEGF or PROK1 protein increased significantly in the cases with positive lymph node metastasis, positive hematogenous metastasis, and advanced-stage tumors, suggesting an association between these 2 proteins and malignancy. Furthermore, the recurrence rate was significantly higher when both proteins were expressed in the primary lesions of stage III colorectal cancer patients with a high risk of recurrence when compared with other cases. The prognosis of patients who expressed both proteins was also significantly poorer, thus reflecting the recurrence rate. These 2 proteins are considered important in light of their close relationships with human colorectal cancer metastasis and recurrence.

Regarding the VEGF signaling pathway, VEGF binds to 2 receptors on the cell surface, VEGFR-1 and VEGFR-2, although VEGF signaling primarily acts via VEGFR-2 [33–35]. A dimer forms upon VEGF binding to VEGFR -1 or VEGFR-2, and after autophosphorylation, a cell proliferation-promoting signal is transmitted to the nucleus via a mitogen-activated protein kinase (MAPK) signaling cascade [36]. As a result, angiogenesis occurs and cancer cells use the supplied nutrition and oxygen to support further growth.

Meanwhile, PROK1 signals through the G-protein-coupled receptors prokineticin receptor-1 (PROKR1) and prokineticin receptor-2 (PROKR2) and activates the downstream processes of intracellular calcium flux, p44/p42 MAPK phosphorylation, and the serine-threonine kinase Akt to mediate cellular function [37]. Overall, PROK1 is considered an important factor in cell proliferation, anti-apoptosis, differentiation, and cellular kinetics regulation.

As discussed above, VEGF and PROK1 induce angiogenesis via different cellular signaling pathways that initiate from different receptors. Therefore, these proteins are likely to have different functions. According to the results of a multivariate analysis based on a Cox proportional hazards model, the expression of both PROK1 and VEGF was found to be an independent prognosis factor.

In human colorectal cancers, PROK1 and VEGF are important factors for invasion and metastasis. The combined expression of both proteins comprises a significant prognosis factor, as the prognosis was poor when both proteins were expressed.

These factors are considered to be a good indicator for recurrence rate and prognosis. Also it is expected that the prognosis will be improved for patients that expressed both PROK1 and VEGF by treating chemotherapy and additional therapeutics.

## MATERIALS AND METHODS

### Patients and samples

Surgical specimens and adjacent normal colorectal tissues were obtained from sporadic 620 patients with primary colorectal cancer. Patients who had undergone surgery for primary colorectal cancer at the First Department of Surgery, University of Fukui, Japan between 1990 and 2007, were studied. Tumors were staged according to the TMN classification [38], 117, 199, 223, and 81 were I, II, III, and IV respectively. Histopathological and prognostic findings were evaluated by two pathologists based upon internationally established criteria. Surgical specimens were fixed in 10% paraformaldehyde(pH6.8) for 24 h, and embedded in paraffin.

The eligibility criteria were as follows: (i) a histopathological findings confirmed primary colorectal cancer, (ii) resection of colorectal cancer with extended (D2 or D3) lymphnode dissection [39], (iii) histological curative resection(StageI∼III), (iv) an Eastern Cooperative Oncology Group performance status(PS) of 0 or 1, (v) no chemotherapy or radiotherapy before surgical resection, (vi) Patients with stage III/IV received 5-fluorouracil-based chemotherapy after surgical resection, (vii) Patients with stage I/II received no chemotherapy after surgical resection, (viii) All patients were followed up for recurrence at regular intervals for five years, underwent chest X-ray, computed tomography, and colonoscopy.

### Immunohistochemical study

Paraffin sections were cut 4 μm thick and deparaffinized with xylene. Deparaffinied sections were incubated with 1% hydrogen peroxidase in methanol for 30 minites. The slides were incubated with anti-PROK1 mAb [40] and anti-VEGF mAb(Santa Cruz Biotechnology, USA) for 1 hour. After washing with TBS, staining was analyzed by using for the ChemMate method(Dako, Denmark). Counterstaining was performed with hematoxylin. We decided the cut-off more than 30% of the tumor cells this time. Also we decided the cut-off more than 10% of the tumor cells for VEGF expression. Histopathological diagnosis was evaluated by two pathologists.

### Statistical-analysis

The statistical significance was determined by the χ^2^ test or student *t*-test using Stat Mate IV(ATMS Co., Ltd., Japan).

Survival curves of the patients was performed using the Kaplan-Meier technique. The outcomes from different groups of patients were compared by log rank test using Stat Mate IV(ATMS Co., Ltd., Japan).

The multivariate analysis for patient prognosis was determined by Cox proportional hazards model using SPSS soft ware(IBMM SPSS Statistics, IBM Corporation, USA). Differences were considered significant at *P* values less than .05.
